# The frozen section trap of minimal atypia in synchronous medullary and papillary thyroid carcinoma: A case report

**DOI:** 10.1097/MD.0000000000045628

**Published:** 2025-10-31

**Authors:** Yuanqi Chu, Ming Huang, Jiali Liu, Ru Wang, Xiaojun Xie

**Affiliations:** a Department of Pathology, XD Group Hospital, Shaanxi, Xi’an, China.

**Keywords:** intraoperative frozen section, medullary thyroid carcinoma, papillary thyroid carcinoma, synchronous double primary cancers

## Abstract

**Rationale:**

Synchronous double primary cancer refers to 2 independent primary tumors that occur in the same organ at the same time or within 6 months. We report a case of double primary carcinoma of thyroid gland with different histological subtypes, which is composed of papillary thyroid carcinoma and medullary thyroid carcinoma. Usually, thyroid carcinoma will be confirmed by intraoperative freezing, but it is very easy to miss the diagnosis because of the minimal atypia of medullary carcinoma. This paper introduces a detailed case, aiming to understand the significance of laboratory examination and imaging examination for a clear diagnosis in this case.

**Patient concerns:**

Here, we report a 61-year-old female patient who was found with multiple thyroid nodules by physical examination a month ago. The results of preoperative laboratory examination and puncture biopsy suggested that medullary thyroid carcinoma was possible, but the results of imaging examination and intraoperative frozen section suggested that papillary thyroid carcinoma was possible. In the last routine paraffin section, it was confirmed that it was a case of double primary thyroid carcinoma.

**Diagnoses:**

Paraffin sections of surgical specimens were finally diagnosed as synchronous double primary cancers with different histological subtypes of thyroid.

**Interventions:**

After the operation, the left thyroid gland was fully taken and made, and the final diagnosis was made clear.

**Outcomes:**

The patient has successfully undergone surgery, and the serum calcitonin levels have returned to normal.

**Lessons:**

This rare situation emphasizes the support of imaging examination and laboratory examination for final diagnosis and the limitation of frozen section.

## 1. Introduction

Thyroid cancer is one of the most common head and neck tumors, and its incidence has increased significantly in the past decades, but its growth trend depends on histological subtypes and geographical location. Papillary thyroid carcinoma (PTC) is the most common thyroid malignant tumor, accounting for about 90% of all thyroid cancers.^[1]^ The increase of its incidence rate is mainly attributed to the popularization of ultrasonic screening, and individuals with a diameter <1 cm are called papillary thyroid microcarcinoma. Medullary thyroid carcinoma (MTC) is a rare neuroendocrine tumor, which originates from C cells beside thyroid follicles and is often accompanied by the increase of serum calcitonin, accounting for about 3% to 5% of all thyroid cancers.^[2]^

Usually, 2 kinds of tumors do not occur at the same time, but even so, pathologists should be careful when taking materials. Synchronous double primary cancer refers to 2 independent primary tumors that occur in the same organ at the same time or within 6 months.^[3]^ We report a case of synchronous double primary cancer in thyroid gland, which is even rarer. This kind of synchronous double primary cancer is called double primary cancer with different histological subtypes, and the incidence of this situation is about 0.28%.^[4]^ There have been some case reports and some multicenter studies in the past,^[3,5–9]^ but these reports have not discussed the missed diagnosis of intraoperative freezing due to the small atypia of medullary cancer.

## 2. Case report

The patient, a 61-year-old female, was found to have multiple thyroid nodules by physical examination 1 month ago, and had no symptoms such as neck pain, hoarseness, and dysphagia. One week later, thyroid B-ultrasound examination in other hospitals revealed solid nodules in the middle and lower right lobe (TI-RADS 4A), solid nodules in the middle left lobe with calcification (TI-RADS 4A), and enlarged lymph nodes in the third region of the left neck. Two weeks ago, the patient was diagnosed as MTC by ultrasound-guided puncture biopsy of thyroid nodule in another hospital, which revealed tumor lesions and combined with the increase of serum calcitonin (36.84 pg/mL). Then, in another hospital, B-ultrasound reexamination showed that the nodules in the middle left lobe and the lower right lobe were TI-RADS 5, and PTC was considered. The results of preoperative laboratory examination showed that calcitonin was 36.84 pg/mL (↑), procalcitonin was 0.235 ng/mL (↑), α-fetoprotein, carcinoembryonic antigen (CEA), CA199, and CA125 were all normal. CT examination: nodules in the middle of left lobe (5 mm) and nodules in the middle of right lobe (6 mm).

**Figure 1.**
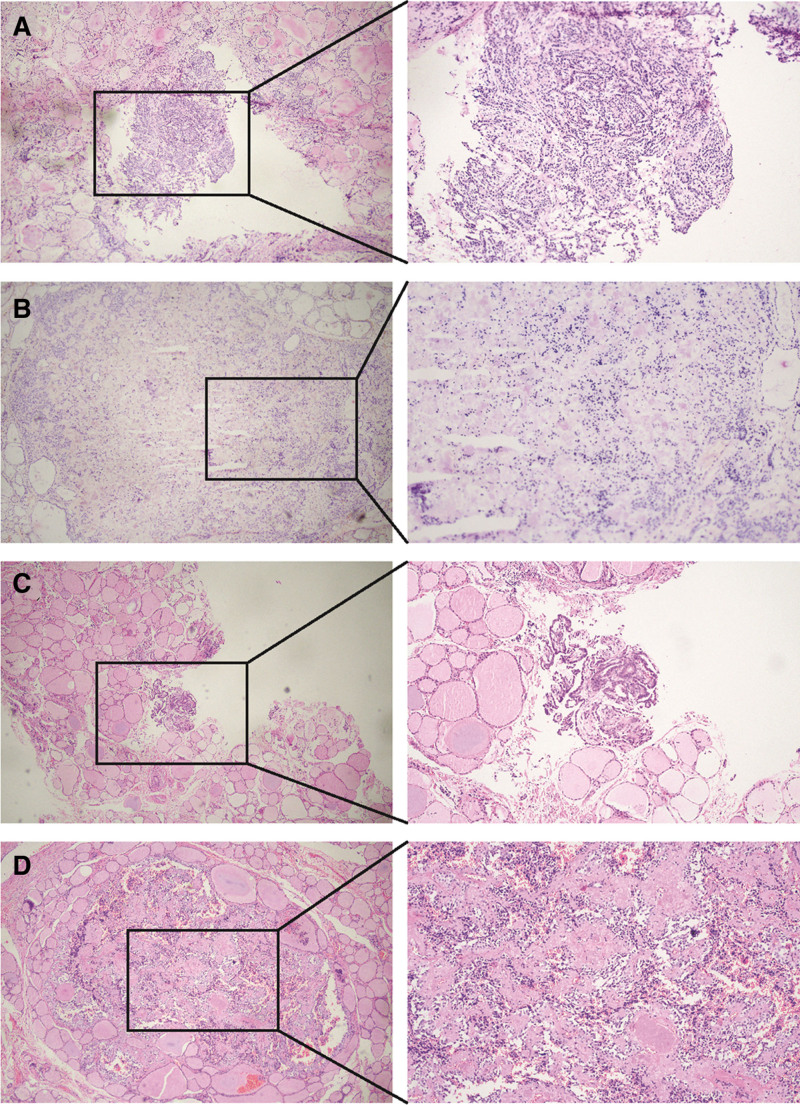
Intraoperative frozen section results. (A) Left thyroid frozen section; (B) right thyroid frozen section. (C) Left thyroid HE section; (D) right thyroid HE section. HE = hematoxylin-eosin staining.

According to the results of the examination, the patient underwent total thyroidectomy and bilateral central lymph node dissection. The frozen pathological report during the operation: nodular goiter on the left and PTC on the right (Fig. 1A).

**Figure 2.**
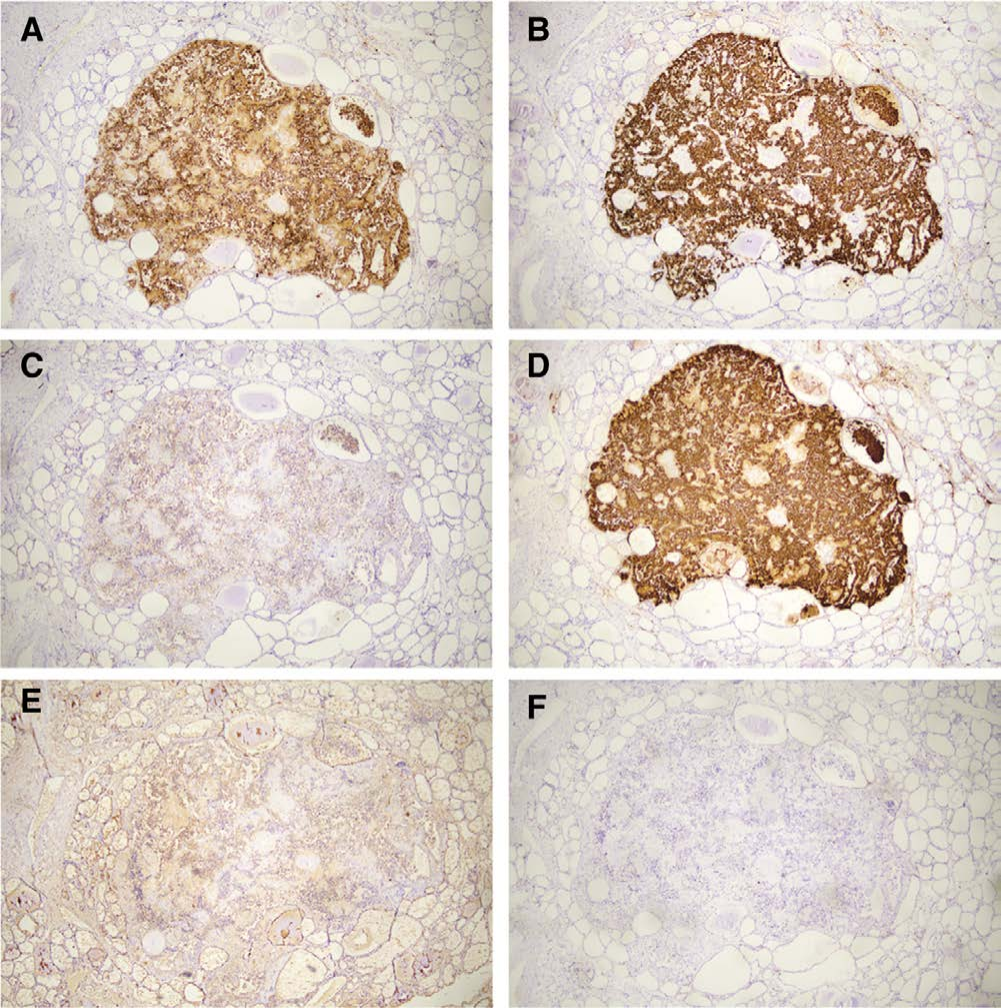
IHC of right thyroid. (A) CgA, (B) Syn, (C) CD56, (D) CEA, (E) TG, (F) TTF-1. CEA = carcinoembryonic antigen.

The result of intraoperative frozen section is not consistent with the previous results of puncture biopsy in other hospitals and the increase of calcitonin in patients. After routine paraffin staining, a tiny nodule with a diameter of about 0.5 cm can be seen by naked eyes, which is composed of a group of mild round and polygonal cells with granular cytoplasm and phagocytosis, accompanied by hyaline collagen and amyloid deposition (Fig. 1D). Because of the low cell atypia, medullary carcinoma can not be diagnosed clearly in frozen section (Fig. 1B). The thyroid gland on the right can be clearly defined as PTC (Fig. 1C), and the left thyroid gland is confirmed as MTC by immunohistochemistry: CgA(+), Syn(+), CD56(+), CEA(+), TG(+), TTF-1(−) (Fig. 2A–F). After operation, serum calcitonin decreased to 1.13 pg/mL (normal) and procalcitonin was 0.045 ng/mL (normal).

*Final diagnosis*: synchronous double primary carcinoma with different histological subtypes of thyroid (left thyroid micropapillary carcinoma and right thyroid micropapillary carcinoma).

## 3. Discussion

PTC and MTC originated from thyroid follicular epithelial cells and parafollicular C cells, respectively. Microscopically, PTC showed that tumor cells with ground-glass nuclei, nuclear sulcus and nuclear inclusions formed a true papillary structure. MTC has many cytological features, such as eccentric nucleus, salt and pepper-like chromatin, inconspicuous nucleoli, binuclear or polynuclear, and unclear cytoplasm boundary.^[10]^ The co-occurrence rate of these 2 different histological types of thyroid cancer is only 0.28%, which is usually called synchronous double primary cancer with different histological subtypes, and is classified as mixed medullary–papillary cancer in the classification of the 5th edition of WHO.^[11]^ Although there is still some controversy about the common occurrence of the 2 cancers,^[12]^ most scholars support the collision theory, that is, the occurrence of the 2 cancers is independent, and the simultaneous occurrence of the 2 cancers is accidental.^[13]^] While synchronous MTC and PTC has been reported, our case is distinctive due to the profound diagnostic challenge posed by the nondiagnostic intraoperative frozen section and the conflicting preoperative findings.

Previous reports have described combined tumors, but the critical limitation of frozen section in this context, particularly for small, low-grade MTC components, has not been extensively discussed. Our case provides a stark illustration of this pitfall. The results of preoperative examination and intraoperative frozen section were inconsistent, and finally the patient was diagnosed as synchronous double primary thyroid cancer (MTC combined with PTC) by postoperative pathology and immunohistochemistry. Its diagnosis and treatment process have important clinical implications.

### 3.1. The challenge of preoperative diagnosis: the complementary role of imaging and serum markers

The ultrasonographic features of MTC and PTC partially overlap, but MTC lacks the typical microcalcifications and irregular edges of PTC, and TI-RADS classification has low specificity.^[14]^ In this case, the preoperative ultrasound showed TI-RADS 4A (left lobe) and 5 types (right lobe) respectively, but the final pathology confirmed that the left lobe was MTC and the right lobe was PTC, indicating that MTC might be missed only by relying on imaging, especially when the lesion was small (<1 cm). The detection of serum calcitonin plays a key role in this case. Although preoperative imaging suggested PTC was possible, the increase of calcitonin (36.84 pg/mL) strongly suggested MTC was possible. Therefore, for patients with thyroid nodules, especially multiple nodules, routine detection of calcitonin is helpful to improve the early diagnosis rate of MTC. In addition, if calcitonin is elevated but the puncture results do not support MTC, multipoint puncture or intraoperative freezing combined with calcitonin detection should be considered to reduce missed diagnosis.

### 3.2. Limitations of intraoperative frozen pathology and coping strategies

In this case, only the right PTC was reported during intraoperative freezing, while the left nodule was misjudged as nodular goiter, and MTC was not diagnosed until paraffin section and immunohistochemistry after operation. This phenomenon reflects the difficulty of MTC in the diagnosis of frozen section, and the main reasons include:

*Low cell atypia*: MTC cells are mild in morphology and easily confused with follicular epithelium or colloid. Amyloid is easily overlooked: amyloid deposition in MTC stroma may be misjudged as colloid in frozen sections, and Congo red staining can assist in the identification, but it needs to be considered in advance.When the results of intraoperative frozen section are not consistent with the results of preoperative puncture biopsy in other hospitals, samples should be fully taken and examined after operation. For patients with elevated calcitonin, even if there is no clear evidence of malignancy in frozen sections, the range of samples should be expanded, especially in calcified or hard areas. Rapid calcitonin detection (such as the improved application of iPTH detector) can be considered during operation to assist in judging whether MTC exists. Close communication between pathology department and clinical department is very important. If MTC is highly suspected in clinic but frozen negative, the suspicious area should be marked for further examination after operation.

### 3.3. Treatment plan and follow-up

Total thyroidectomy plus central lymph node dissection is a reasonable choice for this patient, especially MTC is easy to metastasize early (even if the tumor is small). If MTC is known before operation, preventive lateral neck lymph node dissection can be considered (depending on calcitonin level and imaging evaluation). Postoperative follow-up should be monitored separately for 2 kinds of cancers: the follow-up for MTC mainly monitors serum calcitonin and CEA, and if it drops to normal after operation, the prognosis will be better; if it continues to rise, it is necessary to check the residual or metastatic focus. Follow-up for PTC mainly includes monitoring the level of thyroglobulin and neck ultrasound, and taking radioactive iodine therapy when necessary (but MTC is ineffective for iodine therapy).

### 3.4. The value of multidisciplinary collaboration in accurate diagnosis and treatment of thyroid cancer

The diagnosis of this case depends on the close cooperation of ultrasound department, pathology department, endocrine surgery department, and laboratory department, which has different enlightenment for different departments:

*Department of imaging*: for patients with elevated calcitonin, we should be alert to MTC, and pay attention to MTC-related signs such as “hypoechoic, homogeneous and without calcification” when describing the characteristics of nodules.

*Department of pathology*: when the laboratory examination is inconsistent with intraoperative frozen pathology, it is necessary to strengthen communication, expand the materials, and rationally apply immunohistochemistry.

*Department of surgery*: intraoperative decision-making should combine preoperative images, puncture, and serum markers to avoid missing MTC due to false negative freezing.

## 4. Conclusion

This is a case caused by the inconsistency between clinical examination and intraoperative freezing examination. For clinicians, calcitonin detection should be included in the routine evaluation of thyroid nodules, especially for patients with multiple nodules or TI-RADS 4-5 but lacking typical PTC characteristics. MTC is easy to be missed in frozen section, which needs to be combined with clinical, serological and immunohistochemical comprehensive judgment. Double primary thyroid carcinoma (MTC + PTC) is rare but exists, and the biological behavior of both tumors should be taken into account in surgery. In our case, the discrepancy between the preoperative cytology (suggesting MTC), intraoperative frozen section (suggesting PTC/benign), and elevated calcitonin underscored the necessity of MDT. The final diagnosis was only achieved through the integration of pathological reevaluation with permanent sections, correlation with IHC results, and consultation between surgery, endocrinology, and pathology. This directly led to the appropriate surgical management and follow-up plan for both cancers. Future research can explore the molecular mechanism of multiple primary cancers (such as the coexistence mode of RET/RAS/BRAF mutation) and evaluate the cost effectiveness of calcitonin screening to optimize clinical practice.

## Author contributions

**Conceptualization:** Jiali Liu.

**Data curation:** Jiali Liu.

**Formal analysis:** Ming Huang, Jiali Liu.

**Funding acquisition:** Xiaojun Xie.

**Investigation:** Ru Wang.

**Methodology:** Ru Wang.

**Resources:** Yuanqi Chu.

**Software:** Yuanqi Chu.

**Supervision:** Ming Huang.

**Validation:** Ming Huang.

**Visualization:** Ru Wang.

**Writing – original draft:** Yuanqi Chu.

**Writing – review & editing:** Xiaojun Xie.

## References

[1] Fagin JA Longo DL Wells SA. Biologic and clinical perspectives on thyroid cancer. N Engl J Med. 2016;375:1054–67. 27626519 10.1056/NEJMra1501993 PMC5512163

[2] Ball DW. Medullary thyroid cancer: therapeutic targets and molecular markers. Curr Opin Oncol. 2007;19:18–23. 17133107 10.1097/CCO.0b013e32801173ea

[3] Gadong LC Crisostomo T. Simultaneous occurrence of papillary carcinoma and medullary carcinoma. J ASEAN Fed Endocr Soc 2019;34:226–8. 33442161 10.15605/jafes.034.02.16 PMC7784209

[4] Erhamamci S Reyhan M Kocer NE Nursal GN Torun N Yapar AF. Simultaneous occurrence of medullary and differentiated thyroid carcinomas. Report of 4 cases and brief review of the literature. Hell J Nucl Med. 2014;17:148–52. 24997082 10.1967/s002449910137

[5] Wang Y Yin D Ren G Wang Z Kong F. Mixed medullary‑follicular thyroid carcinoma: a case report and literature review. Oncol Lett. 2023;26:429. 37664658 10.3892/ol.2023.14015 PMC10472022

[6] Abdelaal A El Ansari W Abusabeib A Farghaly H Tabeb AAM. Simultaneous occurrence of follicular and papillary thyroid carcinomas in same thyroid lobe: a case series of six patients from Qatar. Int J Surg Case Rep. 2020;73:65–70. 32645594 10.1016/j.ijscr.2020.06.070 PMC7341056

[7] Wong RL Kazaure HS Roman SA Sosa JA. Simultaneous medullary and differentiated thyroid cancer: a population-level analysis of an increasingly common entity. Ann Surg Oncol. 2012;19:2635–42. 22526904 10.1245/s10434-012-2357-8

[8] Appetecchia M Lauretta R Barnabei A. Epidemiology of simultaneous medullary and papillary thyroid carcinomas (MTC/PTC): an italian multicenter study. Cancers. 2019;11:1516. 31600997 10.3390/cancers11101516 PMC6826384

[9] Zhang D Yang M Zhang X. Thirty synchronous medullary and papillary thyroid carcinomas. Front Endocrinol (Lausanne). 2023;14:1153248. 37065753 10.3389/fendo.2023.1153248 PMC10102529

[10] Gild ML Clifton-Bligh RJ Wirth LJ Robinson BG. Medullary thyroid cancer: updates and challenges. Endocr Rev. 2023;44:934–46. 37204852 10.1210/endrev/bnad013 PMC10656709

[11] Bai Y Kakudo K Jung CK. Updates in the pathologic classification of thyroid neoplasms: a review of the world health organization classification. Endocrinol Metab (Seoul, Korea). 2020;35:696–715. 10.3803/EnM.2020.807 PMC7803616 33261309

[12] Ciampi R Romei C Pieruzzi L. Classical point mutations of RET, BRAF and RAS oncogenes are not shared in papillary and medullary thyroid cancer occurring simultaneously in the same gland. J Endocrinol Invest. 2016;40:55–62. 27535135 10.1007/s40618-016-0526-5

[13] Kawasaki K Kai K Tanaka N. Collision tumor of a papillary and follicular thyroid carcinoma: a case report. Thyroid Res. 2023;16:24. 37544981 10.1186/s13044-023-00167-3 PMC10405457

[14] Piciu D Piciu A Fetica B. Thyroid carcinoma associated with other primary neoplasms, a single center study. Med Pharm Rep. 2021;95:275–81. 10.15386/mpr-2346 PMC9387584 36060512

